# The unconventional kinetoplastid kinetochore: from discovery toward functional understanding

**DOI:** 10.1042/BST20160112

**Published:** 2016-10-19

**Authors:** Bungo Akiyoshi

**Affiliations:** Department of Biochemistry, University of Oxford, Oxford, U.K.

**Keywords:** chromosomes, kinetochores, trypanosomes

## Abstract

The kinetochore is the macromolecular protein complex that drives chromosome segregation in eukaryotes. Its most fundamental function is to connect centromeric DNA to dynamic spindle microtubules. Studies in popular model eukaryotes have shown that centromere protein (CENP)-A is critical for DNA-binding, whereas the Ndc80 complex is essential for microtubule-binding. Given their conservation in diverse eukaryotes, it was widely believed that all eukaryotes would utilize these components to make up a core of the kinetochore. However, a recent study identified an unconventional type of kinetochore in evolutionarily distant kinetoplastid species, showing that chromosome segregation can be achieved using a distinct set of proteins. Here, I review the discovery of the two kinetochore systems and discuss how their studies contribute to a better understanding of the eukaryotic chromosome segregation machinery.

## Introduction

**Figure 1.**
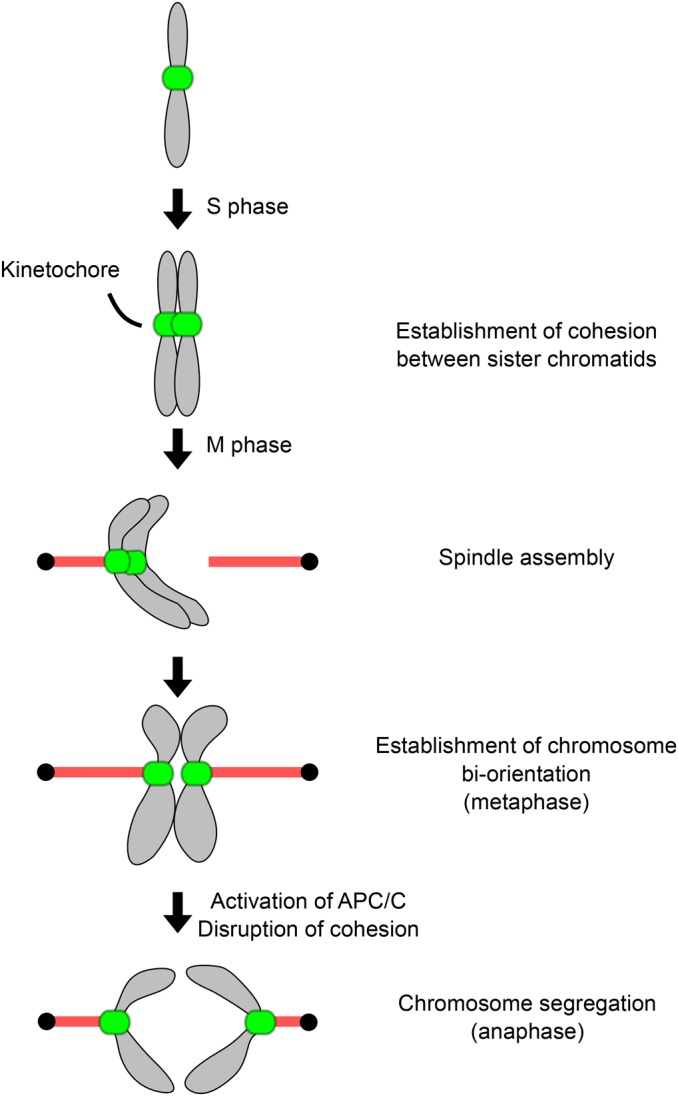
Chromosome segregation. Chromosome duplication occurs during S phase, linking sister chromatids by cohesion. When cells enter mitosis, a bipolar spindle is assembled and kinetochores start to interact with dynamic microtubules. Once all chromosomes achieve bi-oriented attachments, the spindle checkpoint is satisfied. Subsequent activation of APC/C leads to the destruction of cohesion, segregating sister chromatids away from each other in anaphase.

Faithful transmission of genetic information from generation to generation is essential for the survival of all organisms. In eukaryotes, chromosome replication occurs during S phase and duplicated chromosomes are physically connected by the cohesin complex [1] (Figure 1). Chromosome segregation is directed by the kinetochore, the proteinaceous structure that assembles onto the centromeric DNA and interacts with spindle microtubules during mitosis and meiosis [2–5]. In many species, centromeres consist of repetitive sequences that span several kilobases up to megabases [6]. Kinetochores assemble onto a small portion of these sequences and their positional information is epigenetically inherited from generation to generation [7–9]. Microtubules are dynamic polymers that grow and shrink by incorporating or dissociating α-/β-tubulin subunits at their tips [10]. By interacting with microtubules, kinetochores enable duplicated chromosomes to align at the center of the spindle. Accurate segregation requires sister kinetochores to make bi-oriented attachments to microtubules emanating from opposite poles. Attachment errors must be recognized and corrected prior to anaphase [11]. The spindle checkpoint monitors attachment status and delays the onset of anaphase until all chromosomes form correct bi-oriented attachments [12,13]. Once all chromosomes have achieved bi-orientation, the anaphase promoting complex (APC/C) is activated, leading to the cleavage of cohesin complexes and separation of sister chromatids [14–16].

Given the central role of kinetochores in co-ordinating chromosome segregation, elucidating their molecular mechanism of action is a fundamental issue in cell biology. The most basic kinetochore functions are to bind DNA and dynamic microtubules. What kind of proteins carry out these functions? How did they evolve? Identifying and characterizing the constituent proteins are clearly necessary as the first step to address these questions. Here, I review the discovery of two different kinetochore systems and discuss how their studies will contribute to a better understanding of chromosome segregation machines in eukaryotes. I place emphasis on the unconventional kinetoplastid kinetochore and refer readers to comprehensive reviews on the conventional kinetochore [3,4,17–19].

## Conventional kinetochores

### Discovery

Centromeres/kinetochores were first recognized cytologically as primary constrictions on each chromosome where spindle microtubules attach [20]. Kinetochore structures were visualized by electron microscopy [21–23], revealing a trilaminar structure at the periphery of the centromere. Identification of kinetochore components was made possible by the discovery of autoimmune sera from patients with scleroderma spectrum disease that stained the centromere regions of chromosomes [24]. Using the sera, the first kinetochore proteins (centromere proteins, CENP-A, B, C) were recognized [25,26]. Their cDNA clones were subsequently isolated and protein sequences revealed [27–29].

Although the first kinetochore proteins were discovered in humans, many additional kinetochore proteins were subsequently identified in budding yeast owing to the power of genetics and its simple centromere structure [30]. For example, biochemical purifications of centromere DNA-binding proteins [31], genetic screens for mutants that have increased rates of chromosome loss [32,33], one-hybrid [34] and two-hybrid screens [35] all led to the identification of many kinetochore proteins. More recently, affinity purification and mass spectrometry analysis accelerated the identification [36,37].

Increased sensitivity in homology detection algorithms has allowed the identification of homologous proteins in more and more species as the number of sequenced genomes has expanded [38–41]. Genome-wide RNAi screens [42,43] and proteomic analyses of isolated chromosomes [44,45] also identified kinetochore proteins. To date, more than 80 proteins have been identified that at least transiently localize to kinetochore regions, including structural kinetochore proteins (e.g. CENP-A, CCAN, KNL1, Mis12 and Ndc80), regulatory proteins [e.g. Aurora B/chromosomal passenger complex (CPC), Mps1, Plk1, PP1 and PP2A], and spindle checkpoint proteins (e.g. Bub1, Bub3, BubR1/Mad3, Mad1 and Mad2). After identifying proteins that localize at kinetochores, the next crucial step is to reveal their function. Most notably, which proteins bind DNA or microtubules?

### Identification of DNA-binding kinetochore proteins

Cloning and sequencing of CENP-A cDNA revealed that it is a histone H3 variant [29,46]. This was a very exciting finding because it not only revealed that CENP-A is a DNA-binding protein but also suggested a possible mechanism for how kinetochore positions could be epigenetically inherited [47,48]. Subsequent studies have shown that CENP-A is essential for kinetochore assembly in various eukaryotes [42,49,50]. Given its importance in kinetochore biology, extensive studies have been performed to better understand the nature and function of CENP-A ([51–54]).

Other proteins that can bind DNA, at least *in vitro*, include CENP-C^Mif2^ [55,56], CENP-U^Ame1^/CENP-Q^Okp1^ [57], and CENP-T/W/S/X [58–60]. Minor groove DNA-binding motifs are found in some of these proteins: the AT-hook [61] in CENP-C^Mif2^ and CENP-U^Ame1^/CENP-Q^Okp1^ [56,57,62], and the SPKK motif [63] in CENP-A [64]. However, these short motifs are often poorly conserved even among closely related species, suggesting that they are probably not essential for their functions but may contribute to enhancing their DNA-binding affinity [56]. Regardless, the presence of these short motifs could be indicative of DNA-binding activities for a given protein.

### Identification of microtubule-binding kinetochore proteins

Human Ndc80^HEC^ was originally identified in a two-hybrid screen with retinoblastoma protein as bait [65], whereas its yeast homolog was identified in a spindle pole body preparation [66]. Ndc80 interacts with Nuf2, Spc24, and Spc25, which together form a rod-shaped complex [67,68]. All four subunits contain globular domains and extensive coiled-coil regions. Although depletion studies showed that the Ndc80 complex is essential for kinetochore–microtubule attachment [36,69,70], it was not clear whether the complex directly interacts with microtubules or recruits other kinetochore proteins that bind microtubules. It was an *in vitro* microtubule co-sedimentation assay that revealed that the former was the case [71,72]. However, it took nearly a decade since the identification of Ndc80 to assign it a function at the kinetochore. Given its now recognized importance in microtubule-binding, extensive studies have been carried out to mechanistically understand how the Ndc80 complex binds to microtubules ([73–78]).

### Kinetochore subcomplexes and hierarchical assembly

Another important aspect of kinetochore research is to understand its design principles. Electron microscopy studies have visualized the overall architecture of kinetochores within cells [79,80] as well as isolated kinetochores [81]. At the molecular level, kinetochores are composed of biochemically stable/isolatable subcomplexes, such as the Ndc80 subcomplex, Mis12 subcomplex, and CCAN [37,82–86]. It is thought that these subcomplexes assemble in a hierarchical manner at the kinetochore [82]. CENP-A is at the base of this hierarchy and recruits CENP-C [52,87], which in turn recruits the Mis12 subcomplex [88,89] as well as CCAN components [90,91]. The KNL1, Mis12, and Ndc80 subcomplexes can form a larger complex called the KMN network that has microtubule-binding activities [71,92]. The relative positions of these subcomplexes within kinetochores have been determined by super-resolution microscopy, revealing the overall protein architecture of kinetochores [93–95].

### Structural features

The majority of kinetochore proteins lack obvious functional domains or similarity to other proteins at the primary sequence level. However, structural analyses have revealed common domains in proteins that do not have significant sequence similarity. For example, the RWD domain was found in Spc24, Spc25, CENP-P^Ctf19^, CENP-O^Mcm21^, Csm1, Mad1, and KNL1 proteins [67,68,92,96,97], calponin homology domain in Ndc80 and Nuf2 proteins [72,73], and tetratricopeptide repeat domain in Bub1, BubR1, and Mps1 proteins [98,99]. Furthermore, CENP-L^Iml3^ has similarity to the bacterial recombination-associated protein RdgC and TATA-binding protein TBP [100,101], CENP-M folds like a small GTPase [102], the C-terminal dimerization domain of CENP-C^Mif2^ has similarity to the bacterial transcription factor Hth-3 [56], and point centromere-specific Ndc10 has similarity to a bacterial tyrosine recombinase [103,104]. These studies highlight the importance of structural analysis in kinetochore research to reveal distant homology. They also suggest that the highly complicated kinetochores likely originated from a few protein modules aided by gene duplication and functional diversification [105].

### How widely are these kinetochore proteins conserved?

Microtubules appear to be utilized for chromosome segregation in all eukaryotes studied thus far, and α-/β-tubulins are one of the most highly conserved proteins [106–108]. Kinetochore proteins, in contrast, vary to a great extent in primary sequences [109]. Yet, thanks to increased sensitivity in homology search algorithms, we now know that many of the above-mentioned kinetochore proteins are conserved in various eukaryotes [40,62]. CENP-A, CENP-C, and components of the Ndc80 complex are widely found in diverse eukaryotes, suggesting that most eukaryotes utilize these kinetochore proteins to bind DNA or microtubules. However, although CENP-A was thought to be conserved in all eukaryotes, a recent study showed that CENP-A is absent in some holocentric insects [110]. Furthermore, two popular model eukaryotes (*Drosophila melanogaster* and *Caenorhabditis elegans*) appear to have lost the majority of CCAN components [111–114]. Therefore, it has become clear that the repertoire of kinetochore proteins can be dramatically different among eukaryotes despite functional conservation [115].

More extreme cases were found in kinetoplastids, a group of unicellular eukaryotes defined by the presence of kinetoplast which is a large structure in the mitochondrion that contains the mitochondrial DNA [116]. When the genomes of three kinetoplastid species were sequenced (*Trypanosoma brucei*, *Trypanosoma cruzi*, and *Leishmania major*), none of the core structural kinetochore proteins were identified [117–119]. In contrast, components of basic cell cycle machinery were readily identified, including the CDK/cyclin system, the cohesin complex, separase, the condensin complex, Aurora B kinase, Polo-like kinase, MAD2, APC/C, and proteasomes [120]. However, chromosome segregation depends on spindle microtubules [121], and electron microscopy has visualized kinetochore-like electron-dense plaques that appear to form end-on attachments to spindle microtubules in mitotic cells [122–124]. Yet, nothing was known about the identity of kinetochore components in kinetoplastids. Do they have conventional kinetochore proteins that simply diverged too far in primary sequences? Or do they have previously unforeseen types of kinetochore proteins?

## Unconventional kinetochores in kinetoplastids

### Discovery: personal reflections

My scientific career started in 2003 when I joined Dr Yoshinori Watanabe's group in Tokyo to study how Polo-like kinase modulates kinetochore function to promote meiosis-specific chromosome segregation in fission yeast [125]. I then moved to Dr Sue Biggins' laboratory in Seattle to study mitotic kinetochores using budding yeast. There, I achieved the isolation of native kinetochores for the first time [44,126,127] and obtained a PhD. When I was thinking about projects for my post-doctoral training, I became interested in finding out whether basic kinetochore proteins are conserved in all eukaryotes. After I learned that kinetoplastids do not have any apparent canonical kinetochore components including CENP-A [128,129], I joined the laboratory of Dr Keith Gull in Oxford in November 2010 to identify kinetochore proteins in *T. brucei*. There were no magical antibodies that recognized the centromere regions of its chromosomes, so how could I achieve this goal from scratch? With my expertise in protein purification, I first immunoprecipitated conserved mitotic proteins and performed mass spectrometry to identify interacting proteins [the immunoprecipitation (IP)/mass spectrometry (MS) approach]. Because spindle checkpoint components co-purify with kinetochore proteins in other eukaryotes [130], I first made a YFP fusion for MAD2, the only spindle checkpoint protein homolog identified in *T. brucei*. However, this protein turned out to localize at basal bodies, having no detectable signal at kinetochores [120]. Consistent with this observation, IP/MS of YFP-MAD2 only led to the identification of another protein that localized to basal bodies (B. Akiyoshi, unpublished data). I then made YFP fusions for various proteins, including AUK1 (Aurora B kinase homolog [131]), CDC20 (activator of the APC/C [132]), and XMAP215 (microtubule-associated protein [133,134]). Again, although IP/MS of these proteins worked and identified interacting proteins, this approach failed to identify any protein that localized at kinetochores (B. Akiyoshi, unpublished data).

Another idea I had in mind was to carry out a YFP-tagging screen. The *T. brucei* genome has approximately 9000 proteins, which means that I should identify kinetochore proteins if I study all of them. This was a simple and straightforward idea, but I did not know where to start. It was before the development of PCR-based tagging methods in the laboratory [135], meaning that I had to make targeting plasmids for each gene [136]. Coincidentally, Dr Christine Clayton's group published a cell cycle transcriptome analysis of different cell cycle stages in March 2011 in *PLoS ONE* [137]. Impressed by the quality of data, I immediately started tagging uncharacterized genes whose transcript levels were up-regulated in either S phase or mitosis. This approach paid off. Examination of only 30 genes identified one corresponding protein that had a characteristic kinetochore localization pattern: formation of dots in the nucleus during S phase, their alignment on mitotic spindles in metaphase, and close association with spindle poles during late anaphase. This was my first ‘Eureka!’ moment and happened in June 2011. Later I named the protein KKT1 for kinetoplastid kinetochore protein 1. Using KKT1 as bait, I aimed to identify more kinetochore proteins. However, my first IP of YFP-KKT1 completely failed using the same protocol that had worked for those conserved proteins mentioned above (cryolysis using mortar and pestle). It turned out that the YFP-KKT1 signal remained in the detergent-insoluble fraction, so I tried various methods to release it. I eventually found that sonication efficiently solubilized KKT1, and immunoprecipitation from this sample led to the identification of 12 proteins as possible KKT1 interaction partners. YFP-tagging revealed typical kinetochore localization patterns for all of them, which was another moment of big excitement. By continuing the hunt and repeating the IP/MS of these newly identified proteins, I identified 19 proteins by March 2012 (KKT1–19). These proteins all had kinetochore-like localization and their RNAi-mediated knockdowns resulted in chromosome mis-segregation. However, there was a fairly valid criticism I had to face. ‘Are they really kinetochore proteins?’

In other eukaryotes, people have used various approaches to examine kinetochore localization such as immunoelectron microscopy [138], chromatin immunoprecipitation (ChIP) [139], chromosome spreads [140], and co-localization with other known kinetochore proteins [36]. I first tried to perform immunoelectron microscopy to examine whether KKT proteins localize at the kinetochore-like electron-dense plaques [124], but it turned out that the fixation condition completely killed the YFP epitope. Then, I switched my effort to a ChIP approach, although it had a different problem. That is, the resolution of previously mapped centromere positions [141] did not allow me to determine which specific DNA fragments should be examined by PCR or Southern blot. I struggled >1 year at this stage. Eventually, deep sequencing of ChIP samples revealed that KKT2 and KKT3 were specifically enriched in the centromere regions, thereby showing that these KKT proteins are bona fide kinetochore proteins. We first submitted the paper to *Nature*, but it was rejected without review as being uninteresting, which happened to be the same reaction to Bill Earnshaw's paper on the discovery of CENP-A, B, C [26,142]. We next submitted it to *Cell* and it was accepted within 1 month [143].

**Figure 2.**
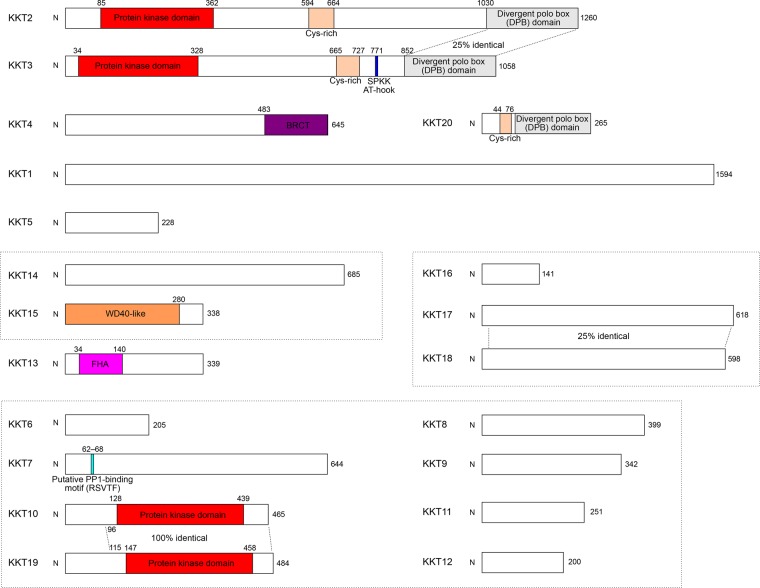
Diagram of *T. brucei* KKT proteins. Identified domains and motifs are shown. Putative subcomplexes are grouped in dotted boxes. Adapted from [143].

I recently identified one more kinetochore protein, KKT20 [144], so 20 KKT proteins have been identified to date (Figure 2). It is important to mention that there may be more kinetochore proteins still waiting to be identified. For example, proteins that weakly or very transiently interact with KKT proteins may well have been missed in the IP/MS approach. Other methods, such as BioID [145] and genome-wide GFP tagging screen, may identify the rest of kinetochore proteins (if any). Another caveat is that any gene not predicted to encode a protein in the *T. brucei* genome database will remain unidentified. With these caveats in mind, I decided to study these KKT proteins in depth.

### Sequence analysis

Initial BLAST and HMMER searches of KKT proteins identified their homologous proteins in both parasitic and free-living kinetoplastids [143]. For most KKT proteins, these searches did not reveal any significant hit outside kinetoplastids. Some KKT proteins had similarity to various proteins due to the presence of the following conserved domains (Figure 2): a BRCT (BRCA1 C terminus) domain in KKT4, an FHA (Forkhead-associated) domain in KKT13, a WD40-like domain in KKT15, and a protein kinase domain in KKT2, KKT3, KKT10, and KKT19. BRCT and FHA are typically protein–protein interaction domains and are often found in proteins involved in the DNA damage response [146]. WD40 is also a protein–protein interaction domain that is one of the most abundant domains in eukaryotic genomes [146]. *T. brucei* has 190 predicted eukaryotic protein kinases, and comparative kinome analysis suggested that KKT2 and KKT3, despite having characteristic features of active protein kinases, do not have a clear affiliation to any known group or family among eukaryotic kinases [147]. Although KKT10 and KKT19 have been classified as members of the CLK/Lammer subfamily in the CMGC family, they may have adapted to carry out kinetochore functions in kinetoplastids as judged by the significant differences between KKT10/KKT19 and the human or *Arabidopsis* CLK/Lammer kinases [143].

I have also been using various sequence analysis software to try to reveal the function and nature of KKT proteins. This includes multiple sequence alignments to determine conserved regions [148], coiled-coil predictions [149], disordered region predictions [150], motif scans [151,152], secondary structure predictions [153,154], and HHpred [155]. Based on these analyses, I recently identified a highly divergent polo box domain (DPB) in the C-terminal region of KKT2, KKT3, and KKT20 [144]. KKT20 does not have a kinase domain, but has similarity to KKT2 and KKT3 in the central domain, suggesting that these proteins likely share common ancestry and that the ancestor might be PLK. In fact, a simple BLAST search showed that the kinase domains of KKT2 and KKT3 are more similar to that of PLK than other kinases. KKT10 and KKT19 exhibit a high degree of similarity at the protein level, as do KKT17 and KKT18. These results suggest that gene duplication played an important role in the evolution of kinetoplastid kinetochores, as seen for conventional kinetochores.

### Predictions of functions

Similar to conventional kinetochores, kinetoplastid kinetochores appear to consist of subcomplexes as judged by the IP/MS results and differential localization patterns [143]. On the basis of IP/MS data, I assigned several putative subcomplexes that could represent functional modules: the KKT14/KKT15 subcomplex, the KKT16/KKT17/KKT18 subcomplex, and the KKT6/KKT7/KKT8/KKT9/KKT10/KKT11/KKT12/KKT19 subcomplex (Figure 2).

**Figure 3.**
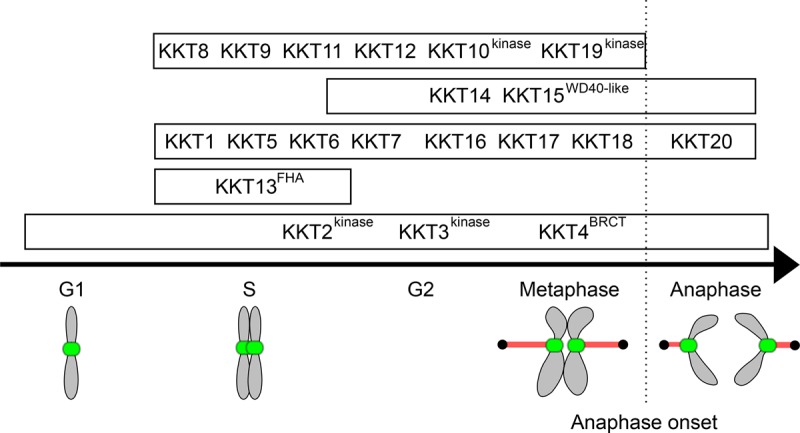
Differential localization timings of KKT proteins. Adapted from [143].

Studies of conventional kinetochore proteins have shown that their functions are often manifested in localization patterns [17]. For example, DNA-binding kinetochore components (e.g. CENP-A and CENP-C) are often constitutively localized at centromeres, whereas microtubule-binding components (e.g. Ndc80 and KNL1) localize to kinetochores specifically during M phase. Although the unconventional kinetoplastid kinetochore proteins may not follow the same principle, there is no evidence to believe otherwise. In fact, the observation that components of the putative subcomplexes mentioned above largely follow the same localization pattern supports this possibility (Figure 3). In this sense, the most promising candidates for DNA-binding proteins are KKT2, KKT3, and KKT4.

### Which KKT proteins bind DNA?

KKT2 and KKT3 have putative DNA-binding motifs (AT-hook and SPKK) in some, but not all, kinetoplastids. This is reminiscent of conventional DNA-binding kinetochore proteins such as CENP-A and CENP-C (see above), supporting the possibility that KKT2 and KKT3 may bind DNA. Besides these motifs, KKT2 and KKT3 have three domains conserved among kinetoplastids: an N-terminal protein kinase domain, a central domain that has conserved Cys residues, and the C-terminal DPB. Expression of truncated proteins *in vivo* can determine which region(s) is important for kinetochore/centromere localization, which might correspond to their DNA-binding domains (e.g. see [55,156,157] for CENP-C studies). It will be necessary to directly test whether KKT2 and KKT3 have any DNA-binding activity *in vitro* using pure proteins. Isolation of native KKT proteins or subcomplexes from trypanosomes in good quantity or stoichiometry turned out to be difficult, at least in my hands. Therefore, recombinant KKT proteins (full length or truncations) will need to be expressed and purified to carry out *in vitro* assays.

While *in vitro* assays are important to dissect their DNA-binding activities, *in vivo* analyses will be essential to determine their relevance. RNAi-mediated knockdown showed that KKT2 is important for faithful chromosome segregation [143], and a kinome-wide RNAi screen showed that KKT3 is essential for growth [158], suggesting that these homologous proteins likely have non-redundant functions. It will be necessary to examine whether other KKT proteins fail to localize to kinetochores in the absence of KKT2 or KKT3 to determine the localization hierarchy. Although inducible RNAi-mediated knockdown experiments can be easily performed in *T. brucei* [159,160], other methods such as conditional knockdowns [161], conditional knockouts [162], or a protein degradation system [163] may be necessary to achieve better depletion efficiency and observe defects.

Besides putative DNA-binding domains, KKT2 and KKT3 also have a protein kinase domain classified as unique among eukaryotic kinases [147]. What are the substrates of these protein kinases? Considering the absence of centromere-specific histones in kinetoplastids [128,164], KKT2/KKT3 might phosphorylate histones specifically at the centromere and this phosphorylation mark might provide centromere identity. Or they might phosphorylate and recruit other KKT proteins onto the kinetochore. Understanding the functions of KKT2 and KKT3 will be key to elucidating the assembly of kinetoplastid kinetochores.

### Which KKT proteins bind microtubules?

Unlike the DNA side, sequence analysis did not reveal any obvious domain implicated in microtubule-binding. In the light of conventional kinetochore research, it will be important to invest efforts to obtain recombinant KKT proteins and perform microtubule co-sedimentation assays. Although tubulins purified from bovine brain are commonly used in sedimentation assays, it may be important to use trypanosome tubulins to detect binding [165,166].

The primary candidates for microtubule-binding kinetochore proteins are the KKT14 and KKT15 proteins whose localization pattern resembles that of Ndc80. It will be crucial to test these and other KKT proteins in a sedimentation assay. Once microtubule-binding proteins are identified, more detailed analyses will need to be carried out to determine whether the mechanism of microtubule-binding is distinct from conventional kinetochore proteins.

### Design principles and regulations

It is also important to reveal the interaction network of KKT proteins. For example, how does the KKT10 kinase localize at kinetochores from S phase and dissociate at the metaphase–anaphase transition? What happens if KKT10 does not dissociate properly? To answer these questions, it will be necessary to reveal which proteins interact with KKT10 and how their interactions are regulated. Numerous phosphorylation sites have been identified on the 20 KKT proteins in my IP/MS analyses (B. Akiyoshi, unpublished data), so it is likely that phosphorylation plays important roles in regulating interactions as in other eukaryotes [167–171]. Although it is challenging to understand the interaction network of 20 proteins, this will be key to gaining insights into the architectural design of kinetoplastid kinetochores.

Aurora B is a highly conserved protein kinase found in all sequenced eukaryotes, including kinetoplastids [172]. It is the catalytic subunit of the CPC that displays dynamic localization patterns during mitosis and regulates various mitotic functions, including kinetochore regulations [173]. As in other eukaryotes, Aurora B localizes to kinetochore regions during prometaphase/metaphase in *T. brucei* ([131] and my unpublished data). Therefore, its kinetochore function might be conserved in kinetoplastids. In line with this possibility, KKT7 has putative-binding motifs for the PP1 phosphatase (RSVTF conserved among kinetoplastids and SILK found in *Leishmania*) that is known to counteract Aurora B activities [174,175].

Aurora B is critical for the destabilization of improper microtubule attachments by phosphorylating kinetochores. It has been proposed that the extent of phosphorylation is dependent on the distance from its substrates, thereby explaining why improper tension-less attachments can be specifically destabilized [176–180]. A key element in this hypothesis is the enrichment of Aurora B at the inner centromere regions, which become physically separated from bi-oriented kinetochores due to microtubule-pulling forces. Indeed, distinct space (∼1 µm) has been observed in between metaphase sister kinetochores in various eukaryotes [181], including yeasts [182], humans [183], plants [184], *Cyanidioschyzon merolae* [185], and *Plasmodium* [186]. In contrast, there is no evidence for such space in kinetoplastids. Electron microscopy studies showed that when sister kinetochore pairs interact with microtubules from opposite poles, they have a back-to-back configuration without distinct space between the two structures in *T. brucei*, *T. cruzi*, and *Leishmania* [123,124,187]. The analysis of YFP-tagged KKT proteins in *T. brucei* supports these observations [143], so Aurora B may never be significantly separated from sister kinetochores in kinetoplastids. Understanding their Aurora B regulation might provide novel insights into the mechanism of chromosome bi-orientation. Interestingly, a recent study in budding yeast showed that its chromosomes can bi-orient without the inner centromere localization of Aurora B [188]. These observations imply that there is still a lot to learn about the molecular mechanism of chromosome segregation in eukaryotes.

The spindle checkpoint is a surveillance mechanism that ensures high fidelity chromosome segregation in eukaryotes [13,189]. Although widely conserved among diverse eukaryotes [190,191], the spindle checkpoint is dispensable for the survival of yeasts, flies, and even human cells under certain conditions [192–196]. Indeed, checkpoint-independent mechanisms are also important for the regulation of the APC/C activities [197–201]. Interestingly, there is no evidence of functional spindle checkpoint or feedback control in kinetoplastids. For example, disruption of mitotic spindles or inhibition of DNA replication do not noticeably delay the onset of anaphase or cytokinesis in *T. brucei* [120,202,203]. Nonetheless, KKT4 and KKT20 co-purified with significant amounts of APC/C components [143,144], and the BRCT domain of a human microcephalin (MCPH1) protein has been shown to interact with the APC/C, at least *in vitro* [204]. Therefore, KKT4 and KKT20 might directly affect the activation/inactivation of the APC/C, regulating anaphase onset in a feedforward manner.

### Which organisms have unconventional kinetochores?

Kinetoplastids are a widespread group of unicellular eukaryotes found in diverse environmental conditions, including hydrothermal vents [205–208]. They are defined by the presence of kinetoplast, a large structure in the mitochondrion that contains the mitochondrial DNA [116]. Kinetoplastids can be divided into two subclasses, the early diverging Prokinetoplastina and the Metakinetoplastina [209]. The latter can be further divided into three orders for the bodonid species (Eubodonida, Parabodonida, and Neobodonida) and the trypanosomatids [210]. KKT proteins are found in all sequenced bodonids (*Bodo saltans* and *Trypanoplasma borreli*) [211] and trypanosomatids (e.g. *T. brucei*, *T. cruzi*, *Leishmania*, and *Phytomonas* spp.) [117–119,212]. Furthermore, at least some KKT proteins are present in an early diverging Prokinetoplastina, *Perkinsela* sp. [213], suggesting that KKT proteins are likely a conserved feature of all kinetoplastids (my unpublished data).

Kinetoplastids belong to Euglenozoa, which is a large and diverse group of flagellates [214]. Four lineages are currently known: Kinetoplastids, Euglenids, Diplonemids, and Symbiontids [215]. Genome sequences are available only for kinetoplastids, and it was not known whether KKT proteins are present specifically in kinetoplastids or common among Euglenozoa [143]. Interestingly, examination of *Euglena gracilis* transcriptome [216] revealed clear homologs of conventional kinetochore proteins, including Ndc80, Nuf2, Spc25, and several candidates for CENP-A (based on the presence of longer loop 1 [217]), as well as spindle checkpoint proteins (Mad1, Mad2, and Mad3) (my unpublished data). In contrast, I failed to identify any obvious KKT proteins in this organism. Although its genome sequence will need to confirm it, these data suggest that *E. gracilis* likely has conventional kinetochore proteins, not KKT proteins. If true, it may be appropriate to re-classify Euglenozoa into two separate groups (see below). It will be interesting to examine whether other Euglenozoa species (e.g. *Diplonema papillatum* [218–220] and *Calkinsia aureus* [221]) utilize conventional or unconventional kinetochore proteins.

## Perspectives

Despite the long research on chromosome segregation machinery in eukaryotes, we know little about its evolutionary origins or history. For example, what kind of segregation machinery was used in the last eukaryotic common ancestor (LECA)? The notions that microtubules are used in all eukaryotes studied thus far and that eukaryotes and tubulins likely evolved from Archaea [222–224] suggest that the LECA probably used tubulin-based polymers to drive chromosome segregation. In contrast, there is little trace of prokaryotic traits in kinetochore proteins, making it difficult to reveal what kind of proteins might have been used to bridge between DNA and tubulin-based polymers in this hypothetical eukaryote.

**Figure 4.**
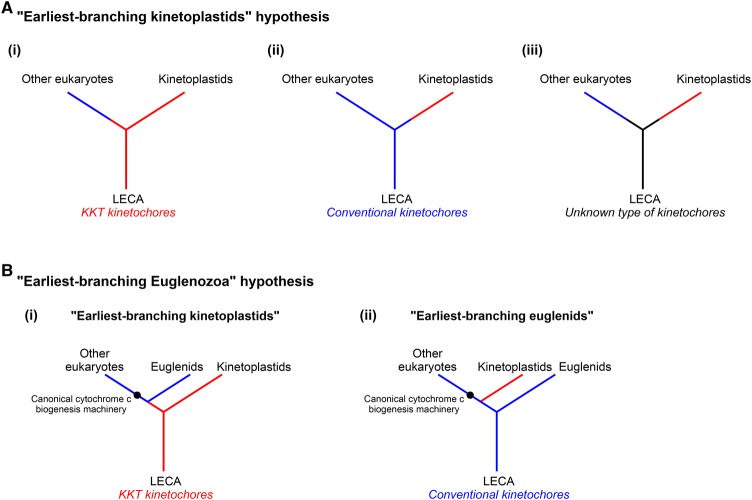
Hypothetical models on early eukaryotic history and origin of kinetoplastid kinetochores. (**A**) The earliest-branching kinetoplastid hypothesis. In this scenario, LECA may have had KKT kinetochores (i), conventional kinetochores (ii), or unknown type of kinetochores (iii). Conventional and KKT-based kinetochores are shown in blue and red, respectively. Note that this is a highly simplified and non-comprehensive set of possibilities of early eukaryotic history and origins of kinetoplastid kinetochores. (**B**) The earliest-branching Euglenozoa hypothesis based on their unique cytochrome *c* biogenesis machinery. In this scenario, either kinetoplastids (i) or euglenids (ii) could be the earliest-branching eukaryotes.

Another difficulty stems from the uncertainty of the position of the root of the eukaryotic tree of life, meaning that we still do not have concrete views about which organisms are the earliest-branching eukaryotes [214,225]. Therefore, it remains unclear whether the unique kinetoplastid kinetochore represents an ancient feature or a derived one. However, among several competing hypotheses [226–228], it has been proposed that Euglenozoa (or species within Euglenozoa) may represent the earliest-branching eukaryotes [229] based on their unique mitochondrial cytochromes *c*/*c1* and the absence of a recognizable biogenesis apparatus for these proteins [230]. The discovery of unconventional kinetochore proteins in kinetoplastids supports this hypothesis. If kinetoplastids are among the earliest-branching eukaryotes (Figure 4A), then the LECA might have possessed the KKT-based kinetochore (Figure 4Ai). In this scenario, only kinetoplastids have retained them while other eukaryotes have lost them and invented conventional kinetochores. Alternatively, it is equally possible that LECA had conventional kinetochores that have been lost in kinetoplastids (Figure 4Aii). It is also possible that LECA had a hitherto unknown type of kinetochores (Figure 4Aiii). Furthermore, the finding that *Euglena* has conventional kinetochore proteins raises the possibility that kinetoplastids, not *Euglena*, could be the earliest-branching eukaryotes, although other interpretations are also possible (Figure 4B). Genome sequences of other Euglenozoa species might provide further insights into this question.

Regardless of the evolutionary origins, understanding the nature of unconventional kinetochore proteins will likely provide important insights into the fundamental principles of eukaryotic segregation machines. Furthermore, the unique kinetochore proteins represent an ideal drug target against parasitic kinetoplastids [231,232]. Understanding the molecular functions of KKT proteins should therefore contribute to developing specific drugs. Finally, it is worth mentioning that there is no organism known so far that contains both types of kinetochores. Can they coexist in a given organism? Is the conventional kinetochore system more accurate than the unconventional one? Are there as-yet different types of kinetochores to be found? Surely a lot of exciting discoveries will be made in the next few decades in the field of chromosome segregation research.

## Abbreviations

APC/C, anaphase promoting complex/cyclosome; CENP, centromere protein; ChIP, chromatin immunoprecipitation; CPC, chromosomal passenger complex; DPB, divergent polo box domain; IP, immunoprecipitation; KKT, kinetoplastid kinetochore; LECA, last eukaryotic common ancestor; MCPH1, microcephalin; MS, mass spectrometry.

## Funding

I am supported by a Sir Henry Dale Fellowship jointly funded by the Wellcome Trust and the Royal Society [grant number 098403/Z/12/Z] as well as a Wellcome-Beit Prize Fellowship [grant number 098403/Z/12/A].
